# Nurses’ Intention to Integrate AI Into Their Practice: Survey Study in Canada

**DOI:** 10.2196/76795

**Published:** 2025-09-05

**Authors:** Guy Pare, Louis Raymond, Faustin Armel Etindele Sosso

**Affiliations:** 1 Research Chair in Digital Health, HEC Montréal, 3000 Cote-Ste-Catherine Road, Montreal, QC, H3T 2A7, Canada, 1 5148828672, 1 5143406812; 2 Institut de recherche sur les PME, Université du Québec à Trois-Rivières, Trois-Rivières, Canada; 3 Université de Montréal, Montreal, QC, Canada

**Keywords:** artificial intelligence, nurses, intention, facilitating conditions, familiarity with AI, beliefs about AI, attitudes toward AI, online survey, mobile phone

## Abstract

**Background:**

The integration of artificial intelligence (AI) into health care is set to revolutionize the sector, offering opportunities to enhance diagnostic accuracy, personalize treatment, and improve patient outcomes. However, little is known about nurses’ readiness to integrate AI into their professional practice.

**Objective:**

This study aimed to identify the key factors influencing nurses’ intention to integrate AI into their practice.

**Methods:**

An online survey was distributed to 3000 members of the professional order of nurses in Quebec, Canada. A total of 312 nurses participated, with 307 completing the full questionnaire. Data were analyzed using descriptive statistics and partial least squares structural equation modeling.

**Results:**

Nurses’ beliefs about the role of AI and trust in AI were found to predict intention to integrate AI significantly. Facilitating conditions influenced beliefs and familiarity with AI, which in turn shaped perceptions of AI’s impact. The model explained 65% of the variance in behavioral intention.

**Conclusions:**

The findings highlight the importance of enhancing nurses’ familiarity with AI and fostering positive beliefs and attitudes to promote effective integration. Educational strategies targeting these beliefs can facilitate AI adoption in nursing.

## Introduction

### Background

The integration of artificial intelligence (AI) into health care is poised to transform the sector by offering unprecedented opportunities to enhance diagnostic accuracy, personalize treatment plans, and improve patient outcomes [1-3]. AI technologies can rapidly and accurately process vast amounts of data, uncovering patterns and generating predictions that may elude human practitioners. These capabilities not only support more accurate diagnoses but also facilitate the development of individualized treatment strategies tailored to each patient’s unique profile, thereby advancing the quality and precision of care delivery.

Beyond reshaping diagnostic and therapeutic processes, AI holds substantial promise for a broad spectrum of health care professionals. For nurses in particular, AI can enhance patient monitoring, reduce workload, and support clinical decision-making in high-stakes tasks such as triage [4]. AI-powered systems can synthesize data from wearable devices, detect early signs of clinical deterioration, and alert nurses in real time, enabling timely and proactive interventions [5,6]. Furthermore, predictive analytics tools can assist with workforce planning by optimizing shift scheduling and staffing levels, helping ensure that nursing resources are allocated efficiently and per patient acuity [7].

AI also opens new avenues for enhancing mental health nursing. For instance, chatbot-based applications and virtual therapists can provide real-time support, extending the reach of mental health services [8]. These tools can analyze linguistic cues in speech and text to detect indicators of depression, anxiety, and other conditions, thereby facilitating earlier identification and intervention by nursing staff [9]. In orthopedic care, AI-driven technologies offer similarly transformative potential. Tools that personalize rehabilitation plans and monitor patient adherence can deliver real-time feedback, promoting the correct execution of exercises and enabling continuous progress tracking [10]. Moreover, AI-enabled robotic assistants and exoskeletons can play a pivotal role in physical rehabilitation by supporting mobility training and functional recovery in patients with musculoskeletal impairments [11].

As health care systems increasingly implement AI technologies [12], it becomes essential to understand the factors that influence nurses’ intentions to integrate these tools into their clinical practice. Although AI holds considerable promise for improving patient care, much of the existing research on AI adoption in health care has focused on physicians and medical students [13,14], leaving the perspectives of other professional groups, particularly nurses, underexplored. This study seeks to address this gap by examining the determinants of nurses’ intention to adopt AI in their practice. Specifically, we investigate the following research question: What factors influence nurses’ intention to integrate AI into their clinical practice? A deeper understanding of these factors will help inform the design of targeted interventions aimed at supporting nurses in the adoption of AI-enabled technologies, thereby enhancing the safety, quality, and efficiency of the health care services they deliver.

The remainder of the paper is structured as follows. We begin by presenting the theoretical foundations that guided the development of our research model and hypotheses. This is followed by a detailed description of the survey design, measurement instruments, and analytical procedures used. We then report the main findings and discuss their implications for nursing education and clinical practice. The paper concludes by outlining the study’s limitations and identifying directions for future research.

### Theoretical Modeling

To address the above-mentioned research question, we first developed a theoretical model grounded in 2 complementary behavioral theories. The first theoretical foundation is the theory of interpersonal behavior (TIB) by Triandis [15], which posits that individuals’ behavioral intentions are shaped by their beliefs about a behavior. In this framework, *beliefs* are understood as cognitive judgments—assessments of what an individual thinks is true or likely regarding a given object, technology, or behavior. These judgments are based on personal perceptions and may or may not reflect objective reality. For example, one may hold the belief that scientific advancements generally contribute to societal well-being. In our study, nurses’ beliefs refer to their perceptions of the role that AI technologies can play in their own professional practice and broader nursing profession, as well as their trust in the reliability and utility of AI-based health technologies. Consistent with prior studies [13,16-19], we argue that more positive beliefs, reflected in stronger perceptions of AI’s professional relevance and greater trust in its outputs, are associated with a higher intention to integrate AI into practice (hypothesis 1 [H1]).

Our second theoretical foundation is the theory of reasoned action (TRA), which emphasizes the role of attitudes as immediate antecedents of behavioral intention [20]. In contrast to beliefs, which are cognitive in nature, attitudes are affective or evaluative responses—feelings of favor or disfavor—toward an object, technology, or behavior. In this study, we operationalize attitudes through 2 constructs: perceived impactfulness of AI (positive) and anxiety toward AI (negative). Perceived impactfulness refers to the extent to which nurses feel that AI-enabled tools can enhance their performance, while AI-related anxiety captures the discomfort, fear, or apprehension experienced when thinking about or interacting with AI-based systems. Prior research suggests that favorable attitudes, such as high perceived usefulness and low anxiety, can promote AI adoption, whereas negative emotional responses may deter it [21-26]. Hence, we posit that nurses with more favorable attitudes toward AI will exhibit a stronger intention to integrate AI into their professional practice (H2).

Both the TIB and TRA highlight that beliefs serve as cognitive precursors to attitudes. That is, individuals form emotional evaluations (ie, attitudes) based on their underlying beliefs about a behavior. For example, Schnall et al [27] found that health care providers’ trust-based beliefs about mobile health technologies influenced their perceived usefulness, an attitudinal judgment. Applying this logic to our model, we hypothesize that nurses with stronger positive beliefs about the role of AI and greater trust in its capabilities will be more likely to develop favorable attitudes toward using AI in their clinical practice (H3).

The third theoretical component of our model centers on “facilitating conditions” as a key determinant of nurses’ behavioral intention to adopt AI technologies. This construct, grounded in the technology acceptance model (TAM)—a foundational framework for examining technology adoption [28]—captures the external factors that influence an individual’s perceived ease of performing a task, such as using AI in clinical settings. In this study, we conceptualize facilitating conditions through two indicators: (1) nurses’ general digital literacy, reflected in their familiarity with consumer technologies such as laptops and mobile phones; and (2) their prior exposure to AI tools, including chatbots and machine learning applications. Building on recent empirical findings by Qaladi et al [29], we posit that greater familiarity with AI fosters a more nuanced understanding of both its capabilities and limitations, which in turn supports more favorable attitudes and intentions toward its adoption in nursing practice.

Prior research suggests that facilitating conditions exert both direct and indirect effects on individuals’ intention to adopt AI technologies. Drawing from the TAM, which posits that external conditions shape beliefs, attitudes, and ultimately behavioral intentions, we hypothesize a similar set of relationships in the context of nursing practice. Specifically, we propose that more favorable facilitating conditions will lead to more positive beliefs about AI among nurses (H4), more favorable attitudes toward AI (H5), and a stronger intention to integrate AI-powered tools into clinical practice (H6).

**Figure 1.**
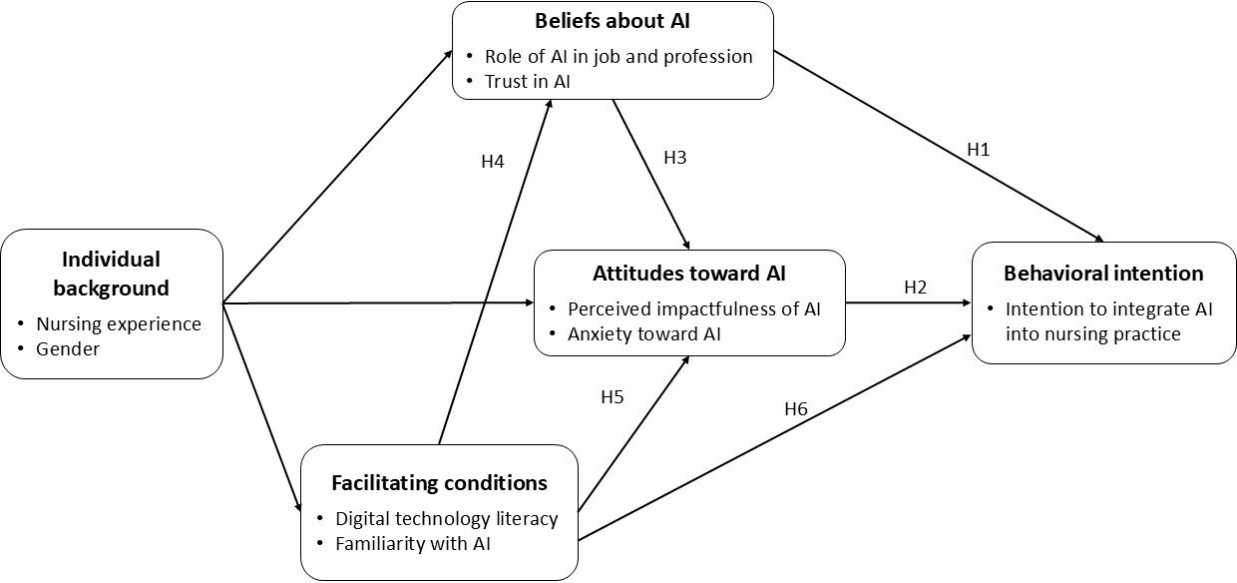
Theoretical model. AI: artificial intelligence.

Additionally, informed by prior studies on digital health training [13,30] and recent applications of the TAM in health care contexts [31,32], we incorporate a fourth construct—individual background—into our research model. This construct is operationalized as a composite of 2 demographic characteristics: gender and years of nursing experience. Given the exploratory nature of this study, we do not posit specific directional hypotheses but rather suggest that individual background may influence nurses’ perceptions of facilitating conditions, their beliefs about AI, and their overall attitudes toward its use. The theoretical model tested in this study is presented in Figure 1.

## Methods

### Study Design and Study Population

This study used a cross-sectional web-based survey design and adhered to the STROBE (Strengthening the Reporting of Observational Studies in Epidemiology) guidelines [33] to ensure methodological rigor and transparency. To obtain a representative sample, we partnered with the professional order of nurses in Québec, Canada. The order randomly selected 3000 active members from its registry of over 82,000 nurses and distributed an invitation letter on our behalf. Eligibility was limited to currently active members, thereby ensuring that respondents were engaged in professional nursing practice. The survey was accessible from the middle of October 2023 to early February 2024, with a follow-up reminder sent 3 weeks after the initial invitation. Participation was voluntary and uncompensated. Respondents accessed the survey via a secure Qualtrics link; the platform complies with relevant data privacy laws and regulations in Canada.

### Questionnaire Development

The operationalization of several research variables included in our model was based on the extant literature. Indeed, given the maturity of the research stream, this approach benefits from previous empirical validations and, thus, ensures the validity and reliability of the selected measurement items. The measures for the dependent variable (behavioral intention) and the role of AI in jobs and professions were adapted from Wagner et al [13], while the measure for trust in AI was adapted from Ouimet et al [34], and the measure for AI anxiety was informed by the work of Wang and Wang [35]. We were unable to locate any pre-existing questionnaire that assessed the perceived impactfulness of AI; hence, we decided to develop our own measure for this construct. Before data collection, we pre-tested the questionnaire with a panel of 4 nursing professionals. Only minor adjustments were made following this validation phase. The measurement items are reported in Multimedia Appendix 1.

### Statistical Analysis

The data were first analyzed through descriptive statistics, using the IBM SPSS software (version 28). Component-based partial least squares structural equation modeling (PLS-SEM) was then used to test the research model (Figure 1), as implemented in the *SEMinR* package (version 2.3.2).

### Ethical Considerations

Ethics approval was obtained from the relevant institutional review board, the HEC Montréal Research Ethics Committee, on August 10, 2023, ensuring that all study procedures adhere to ethical guidelines and standards (#2024‐5512). Before beginning the survey, all participants provided informed consent electronically, having been presented with a comprehensive overview of the study’s objectives, procedures, potential risks, and anticipated benefits. Throughout this study, participant privacy and data integrity were rigorously protected in line with established research ethics guidelines and applicable data protection regulations. No financial incentives or compensation were offered to participants.

## Results

### Overview

A total of 312 nurses responded to the online survey, yielding a response rate of 10%. Of these, 307 respondents completed the full questionnaire and were retained for analysis. As shown in Table 1, the sample included 280 nurses who reported not using AI in their practice (nonusers) and 27 who identified as current users. The respondents were predominantly female, 84.3% (n=236) among nonusers and 74% (n=20) among users, with male respondents comprising 13.6% (38/280) and 22% (6/20), respectively. Regarding professional experience, 55% (154/280) of nonusers and 56% (15/27) of users reported having 16 years or more of nursing experience. Concerning facilitating conditions, AI nonusers reported significantly lower familiarity with AI technologies (mean 3.8, SD 1.9) compared to AI users (mean 5.0, SD 2.1), suggesting that familiarity may play a role in shaping adoption behavior.

**Table 1.** Profile of the respondents.

Nurses’ individual background and facilitating conditions for AI^a^ (VIF)^b^		AI nonusers (n=280)	AI users (n=27)	*t* test; 2-tailed (*df*)	*P* value
Nursing experience (VIF: 1.02; years), n (%)				−0.61 (305)	.55
	1: 0-5	35 (12.5)	3 (11.1)		
	2: 6‐10	51 (18.2)	5 (18.5)		
	3: 11‐15	39 (13.9)	4 (14.8)		
	4: 16‐20	45 (16.1)	4 (14.8)		
	5: 21‐30	70 (25)	3 (11.1)		
	6: 30 or more	39 (13.9)	8 (29.6)		
	Prefer not to respond	1 (0.4)	0 (0)		
Gender (VIF: 1.02), n (%)				1.03 (29)	.31
	1: Female	236 (84.3)	20 (74.1)		
	0: Male	38 (13.6)	6 (22.2)		
	Prefer not to respond	6 (2.1)	1 (3.7)		
Digital technology literacy (VIF: 1.07)				−0.46 (305)	.64
	Mean (SD)	3.7 (0.9)	3.8 (0.8)		
	Minimum	1	3		
	Maximum	5	5		
Familiarity with AI (VIF: 1.07)				−3.01 (305)	.003
	Mean (SD)	3.8 (1.9)	5.0 (2.1)		
	Minimum	1	1		
	Maximum	10	10		

a AI: artificial intelligence.

b VIF: variance inflation factor.

Given the PLS-SEM model to be tested, an a priori power analysis was conducted using G*Power (version 3.1.9.7; Heinrich-Heine-Universität Düsseldorf) [36] to determine the minimum sample size required to detect an *R*² of 0.10 with 80% power and a 5% significance level. Based on this analysis, a minimum of 122 respondents was required [37]. Although response rates of 10% to 15% are common in online social science surveys [38], such rates may introduce the risk of nonresponse bias [39]. To assess this possibility, we first compared the responses of the 61 “late” respondents (ie, the final 20% of participants) with those of the 246 earlier respondents, following the procedure recommended by Hikmet and Chen [40]. No statistically significant differences were found between the 2 groups concerning key variables, including facilitating conditions, beliefs, attitudes, and behavioral intention toward AI. As a second step, we compared the demographic characteristics of our sample with those of the broader population of nurses in Québec. The sample was found to be representative in terms of age, gender, and years of professional experience. Taken together, these 2 assessments suggest that nonresponse bias is unlikely and that our sample reasonably reflects the characteristics of the target population.

### Measurement Model Analysis

**Figure 2.**
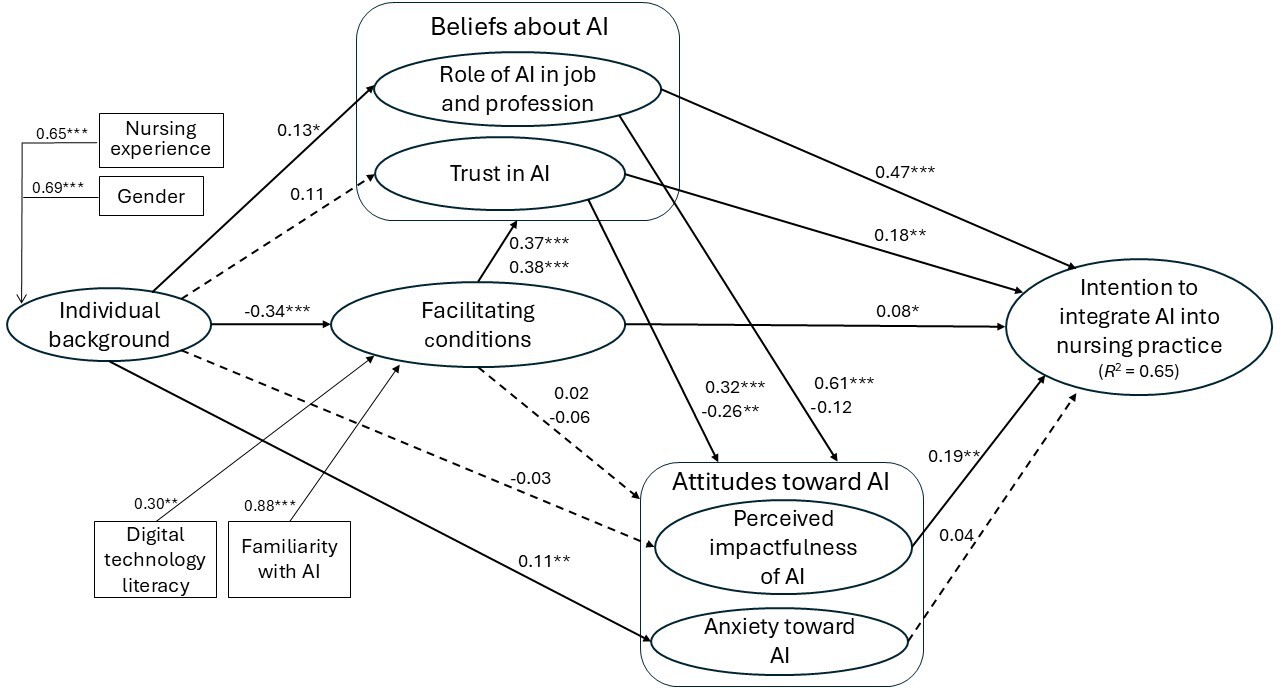
PLS-SEM results (n=280). Gender (0: male; 1: female); * *P*<.10, ** *P*<.05, *** *P*<.001. AI: artificial intelligence; PLS-SEM: partial least squares structural equation modeling.

To address missing data, mean substitution was applied to the research variables included in the analysis. In our model, 2 constructs—individual background and facilitating conditions for AI—are specified as formative, reflecting their composite and multidimensional nature (Figure 2). The remaining 5 constructs are modeled as reflective. The analysis was conducted using the *SEMinR* package for PLS-SEM, which allows for the simultaneous estimation of the measurement and structural models. Accordingly, the psychometric properties of the 7 constructs were assessed within the context of the full research model.

The first step in evaluating the reflective constructs involves assessing their internal consistency and unidimensionality. As shown in Table 2, all Cronbach α values exceeded the recommended threshold of 0.70, indicating satisfactory internal consistency. Unidimensionality was assessed by examining the standardized item (indicator) loadings for each construct. For exploratory research, a loading of at least 0.60 is typically considered acceptable, while a threshold of 0.40 may be tolerated for newly developed scales [37]. The indicator loadings for all reflective constructs met or exceeded these thresholds, thus supporting their unidimensionality.

**Table 2.** Unidimensionality and internal consistency of the research constructs.

Research construct (number of items)	α^a^	Item loadings							
		λ_1_	λ_2_	λ_3_	λ_4_	λ_5_	λ_6_	λ_7_	λ_8_
Role of AI^b^ in jobs and professions (8)	0.89	0.82	0.82	0.87	0.64	0.79	0.61	0.59	0.43
Trust in AI (3)	0.76	0.90	0.82	0.43	N/A^c^	N/A	N/A	N/A	N/A
Perceived impactfulness of AI (6)	0.83	0.85	0.90	0.74	0.90	0.82	0.72	N/A	N/A
Anxiety toward AI^d^	1.00	1.00	N/A	N/A	N/A	N/A	N/A	N/A	N/A
Intention to integrate AI into practice (3)	0.86	0.70	0.95	0.81	N/A	N/A	N/A	N/A	N/A

a Cronbach α coefficient of reliability.

b AI: artificial intelligence.

c N/A: not applicable.

d Index (as opposed to scale) measure.

Next, because the standard reliability and validity criteria used for reflective constructs do not apply to formative constructs, it is essential to assess the absence of multicollinearity among the indicators of each formative construct. This is typically evaluated using the variance inflation factor, with values below 3.3 indicating acceptable levels of collinearity [41]. In our analysis, variance inflation factor values for the 6 formative indicators ranged from 1.01 to 1.29, well within acceptable limits, thereby confirming the absence of problematic multicollinearity.

Once the formative constructs have been validated, we proceeded to assess the reliability, convergent validity, and discriminant validity of the 5 reflective constructs. As shown in Table 3, all composite reliability coefficients exceeded the recommended threshold of 0.70, supporting the internal consistency of these constructs. Evidence of convergent validity is provided by the average variance extracted values, all of which were above the 0.50 threshold, indicating that each construct explains more than half of the variance in its indicators.

**Table 3.** Reliability and validity of the research constructs.

Research construct	cr^a^	AVE^b^	HTMT^c^ matrix						
			1	2	3	4	5	6	7
Individual background	N/A^d^	N/A	N/A	N/A	N/A	N/A	N/A	N/A	N/A
Facilitating conditions for AI^e^	N/A	N/A	N/A	N/A	N/A	N/A	N/A	N/A	N/A
Role of AI in jobs and professions	0.89	0.50	0.27	0.39	N/A	N/A	N/A	N/A	N/A
Trust in AI	0.78	0.56	0.25	0.35	0.76	N/A	N/A	N/A	N/A
Perceived impactfulness of AI	0.93	0.68	0.14	0.31	0.85	0.78	N/A	N/A	N/A
Anxiety toward AI^f^	1.00	1.00	0.29	0.29	0.34	0.37	0.32	N/A	N/A
Intention to integrate AI into practice	0.86	0.68	0.27	0.34	0.77	0.69	0.74	0.26	N/A

a Composite reliability of a reflective construct = (Σλ_i_)2/((Σλ_i_)2+Σ(1−λ_i_2)).

b AVE: average variance extracted (by a construct from its reflective indicators) = Σλ_i_2/n.

c HTMT: heterotrait-monotrait ratio of correlations.

d N/A: not applicable.

e AI: artificial intelligence.

f Index (as opposed to scale) measure.

The final step involved verifying discriminant validity, which ensures that each construct is empirically distinct from the others. For the 2 formative constructs, discriminant validity is supported by the fact that each shares less than 50% of its variance with any other construct (ie, interconstruct correlations remain below 0.71). For the 5 reflective constructs, discriminant validity was assessed using the heterotrait-monotrait ratio of correlations. As indicated in Table 3, all heterotrait-monotrait ratio of correlations values fell below the 0.90 threshold [42], thus confirming satisfactory discriminant validity.

The use of a self-administered questionnaire with a single respondent per case introduces the potential for common method bias (CMB), which can inflate observed relationships among constructs. To mitigate this risk, we implemented procedural remedies, including the use of varied question formats and response scale types. Additionally, we conducted statistical checks to assess the presence of CMB. First, we examined the correlation matrix among the 5 reflective constructs. According to prior recommendations, interconstruct correlations exceeding 0.90 may indicate problematic common method variance [43]. In our data, the highest observed correlation—between role of AI in jobs and professions and perceived impactfulness of AI—was 0.78, suggesting that CMB is unlikely to be a significant concern.

To further assess CMB, we conducted the Harman single-factor test, which involves an exploratory factor analysis of all reflective items without rotation [44]. The results revealed the emergence of multiple factors, with no single factor accounting for the majority of the variance (ie, less than 50% of the total covariance). Taken together, these findings provide reasonable assurance that CMB does not pose a significant threat to the validity of our results.

### Path Analysis

The research model was tested by evaluating the path coefficients (β) that link the constructs in the research model, as shown in Figure 2.

- H1 (confirmed): results from the PLS-SEM analysis indicate a positive effect of nurses’ beliefs about AI on their behavioral intention to adopt it. Specifically, nurses who perceive AI as playing an important role in their jobs and professions report a stronger intention to integrate AI into their practice (β=.47, *P*<.001). Additionally, trust in AI also exerts a significant, though smaller, positive effect on behavioral intention (β=.18, *P*=.0389).
- H2 (partly confirmed): this hypothesis posited a positive relationship between nurses’ attitudes toward AI and their intention to adopt it. This relationship is partially supported by the data. Nurses’ perceptions of AI’s impactfulness are significantly associated with their behavioral intention (β=.19, *P*=.0357). However, their feelings of anxiety toward AI do not significantly influence intention (β=.04, *P*=0.1615).
- H3 (partly confirmed): the expected positive relationship between nurses’ beliefs about the role of AI in their jobs and professions and their attitude toward AI was partly confirmed. A significant positive effect is observed between nurses’ perceptions of AI’s role in their job and their views of its impactfulness (β=.61, *P<*.001), but no significant relationship is found between these beliefs and anxiety about AI (β=−.12, *P*=.1715). In contrast, nurses’ trust in AI significantly influences both dimensions of attitude: positively with perceived impactfulness (β=.32, *P<*.001) and negatively with anxiety (β=−.26, *P*=.0114).
- H4 (confirmed): the analysis reveals that facilitating conditions exert a positive and statistically significant influence on both components of nurses’ beliefs about AI. Specifically, facilitating conditions are positively associated with nurses’ perceptions of AI’s role in their jobs and professions (β=.37, *P*<.001), as well as with their trust in AI (β=.38, *P*<.001). As shown in Figure 2, the most influential explanatory element within the facilitating conditions construct is familiarity with AI, highlighting it as a critical enabler of positive beliefs regarding AI adoption in nursing practice.
- H5 (unconfirmed): contrary to expectations, facilitating conditions—particularly nurses’ familiarity with AI—were not found to significantly influence attitudes toward AI. Specifically, no significant relationship was observed between facilitating conditions and perceptions of AI’s impactfulness (β=.02, *P*=.3194), nor between facilitating conditions and feelings of anxiety related to AI (β=−.06, *P*=.1792). These findings suggest that while facilitating conditions shape beliefs, they do not directly affect attitudes, and thus their influence on behavioral intention is indirect, operating through beliefs rather than attitudes.
- H6 (confirmed): as hypothesized, a statistically significant, though modest, direct effect was found between facilitating conditions and nurses’ behavioral intention to adopt AI (β=.08, *P*=.0599). More importantly, facilitating conditions also exert an indirect effect on intention, mediated through nurses’ beliefs about AI. This finding underscores the importance of ensuring adequate support structures and familiarity with AI to foster both belief formation and intention to adopt AI tools in nursing practice.

Regarding the influence of individual background characteristics, the results indicate that experienced female nurses reported lower levels of familiarity with AI, stronger beliefs about AI’s role in their jobs and professions, and higher levels of anxiety toward AI when compared to inexperienced male nurses. Taken together, nurses’ familiarity with AI, their beliefs about its relevance, and their attitudes toward its use explained 65% of the variance (*R*²) in their intention to integrate AI into clinical practice. This substantial explanatory power provides strong empirical support for the overall validity of the research model tested in this study.

## Discussion

### Principal Findings

This study aimed to identify the key factors influencing nurses’ intention to integrate AI into their clinical practice. Our findings offer several important insights into the relationships among nurses’ familiarity with AI, their cognitive beliefs about AI, their affective attitudes toward AI, and their behavioral intentions regarding its integration in nursing practice.

Our study found that nurses’ beliefs, specifically their perceptions of AI’s relevance to their role and their trust in AI technologies, were significant predictors of their intention to integrate AI into clinical practice. Nurses who recognized the meaningful contribution of AI to the nursing profession and trusted its outputs were more inclined to adopt AI tools. These results are consistent with the TIB [15] and prior research [13,16,17], which emphasize the role of beliefs as cognitive antecedents to behavioral intention.

These findings are further supported by a recent scoping review on AI in health care [45], which identified trust in AI systems and perceived clinical relevance as 2 of the most frequently reported facilitators of adoption among health care professionals. In line with this broader evidence base, our study reinforces the centrality of belief-related factors in shaping adoption intentions. Importantly, the review also highlights a persistent gap in nursing-specific empirical studies, a gap our study addresses by focusing exclusively on nurses and providing actionable recommendations for strengthening both educational and organizational readiness for AI adoption and integration.

Second, while attitudes also played a role, their influence was more modest. Nurses’ perceptions of AI’s impactfulness, representing a positive evaluative attitude, were positively associated with intention, although the effect size was weaker than that of beliefs. In contrast, AI-related anxiety, a negative affective attitude, did not significantly predict behavioral intention. This lack of association may be explained in part by the relatively low levels of anxiety reported by participants (mean 0.9, SD 1.3 on a 0‐4 scale). These findings suggest that although attitudes matter, they may be secondary to beliefs in predicting the intention to adopt AI. This pattern aligns with prior evidence in behavioral science, where cognitive beliefs often explain more variance in intention than affective evaluations alone [46,47].

Third, our results also highlight the importance of facilitating conditions, particularly familiarity with AI, in shaping beliefs. Nurses with greater exposure to and understanding of AI technologies were more likely to hold positive beliefs about AI’s professional relevance and to trust AI-based tools. However, these conditions did not significantly shape nurses’ attitudes, either in terms of perceived impactfulness or anxiety. This suggests that familiarity with AI serves as a foundational enabler of belief formation, which in turn influences attitudes and, ultimately, intention.

Finally, consistent with the TRA [20], we found that beliefs not only had a direct effect on intention but also indirectly influenced intention through their effect on attitudes. This mediating role reinforces the sequential logic of our model, wherein facilitating conditions act as antecedents to beliefs, beliefs act as antecedents to attitudes, and both beliefs and attitudes contribute to the formation of behavioral intention with regard to integrating AI into nursing practice.

### Study Implications

Our study contributes to the growing body of literature on AI integration in nursing by providing empirical evidence on the factors influencing nurses’ intentions to adopt AI. As health care services and systems evolve from physician-centric models toward greater interdisciplinarity [47,48], our findings help fill an important gap by focusing on the beliefs, attitudes, and intentions of nursing professionals, an understudied group in AI adoption research.

Our results have 3 main implications for nursing education, professional development, and institutional policy. First, the strong influence of familiarity with AI on both beliefs and behavioral intentions underscores the importance of targeted efforts to build AI literacy. It is important to distinguish here between general digital technology literacy, defined in our study as familiarity with everyday consumer technologies (eg, smartphones and mobile apps), and nursing informatics (NI) competencies, which encompass more advanced skills such as data interpretation, digital communication, and decision-making support using AI.

In Canada, recent updates to the national NI competency framework reflect this shift by explicitly including AI-related competencies that nursing students and professionals are expected to develop. These include understanding AI applications in clinical contexts, recognizing their limitations, and collaborating with AI-driven decision support systems. Raising awareness of these updated expectations is essential, particularly among nurse educators, curriculum designers, and clinical leaders. As noted by Lattuca et al [48], the novelty and rapid evolution of AI in health care mean that many educators and practitioners are still unfamiliar with how to prepare nurses to engage with these technologies.

To address this gap, we recommend that undergraduate and graduate nursing curricula incorporate dedicated AI literacy modules. These modules should go beyond technical content to include ethical considerations, human-AI collaboration scenarios, and critical appraisal of AI-driven decision support systems. Case-based learning and interdisciplinary projects could further support students in developing both conceptual understanding and practical readiness.

Second, for nurses currently in practice, continuing professional development programs should integrate hands-on demonstrations and simulation-based training with real or prototype AI-enabled tools. Providing opportunities to interact with AI in low-risk environments can enhance understanding, reduce anxiety, and strengthen trust. These interventions are especially important for nurses with less direct exposure to AI and can be aligned with evolving national competency frameworks and accreditation standards.

Third, health care institutions have a role to play in fostering and facilitating conditions for AI integration. This includes providing time and resources for training, supporting access to AI-enabled tools, and designating clinical champions who can help guide and mentor peers in adopting these technologies. Creating such conditions not only supports individual readiness but also helps build organizational capacity for responsible and effective AI deployment in nursing care.

### Study Limitations and Future Research

This study has several limitations that should be acknowledged. First, the cross-sectional design of this study limits our ability to draw causal inferences. While our model is grounded in established behavioral theories, we cannot ascertain the temporal ordering of the observed relationships. Future research should consider longitudinal designs that track changes in nurses’ beliefs, attitudes, and behavioral intentions over time. For instance, studies could follow nurses before and after targeted AI training programs or throughout AI implementation in clinical settings. This would allow researchers to assess whether increased exposure to AI technologies results in durable shifts in intention and eventual adoption. Longitudinal studies would also enable examination of organizational learning mechanisms and help identify critical windows for intervention.

Second, although our sample was shown to be demographically representative of the broader population of nurses in Québec, the response rate of 10% remains a potential limit. While this rate is consistent with prior online survey research targeting health care professionals [38], it may still introduce concerns regarding nonresponse bias. Despite conducting 2 standard tests, we acknowledge that our sample may underrepresent nurses with lower interest in or exposure to AI. Consequently, the relationships observed in our model (eg, between familiarity with AI and intention to integrate AI) may be somewhat inflated due to self-selection bias. Future studies using complementary methods (eg, follow-up interviews, alternative sampling frames) could help confirm the robustness and generalizability of these findings.

Third, a further limitation lies in our operationalization of familiarity with AI, which was measured using a single self-assessment item. While this approach offers a simple and scalable indicator, its breadth may result in variability in interpretation. Future studies could enhance content and construct validity by including behavioral indicators (eg, prior use of AI tools such as predictive analytics, chatbots, or clinical decision support systems) or by asking respondents to provide examples of specific AI applications they have encountered. Open-ended questions or mixed-method designs could also enrich understanding of the depth and context of nurses’ familiarity with AI.

Fourth, this study was conducted in a country with an advanced national health system, Canada, which may limit the generalizability of the findings to other national contexts. Future studies could replicate this research in different countries and health care systems to compare results and enhance generalizability.

Fifth, further research is needed to explore other potential factors influencing AI adoption, such as organizational support, peer influence, and regulatory environments. Understanding these factors could provide a more comprehensive picture of the drivers and barriers to AI integration in nursing care.

### Conclusions

Our study highlights the critical role of nurses’ familiarity with AI, beliefs about AI, and attitudes toward AI in shaping their intentions to integrate AI into their practice. By addressing these factors through targeted educational programs and initiatives, health care organizations can promote the adoption of AI-based health technologies, ultimately enhancing the safety, quality, and efficiency of nursing care delivery. NI researchers should continue to investigate the complex interplay of factors influencing AI adoption to inform effective strategies for integrating AI into nursing practice.

## Supplementary material

10.2196/76795 Multimedia Appendix 1 Questionnaire survey items.

## Abbreviations

AI: artificial intelligence
CMB: common method bias
NI: nursing informatics
PLS-SEM: partial least squares structural equation modeling
STROBE: Strengthening the Reporting of Observational Studies in Epidemiology
TAM: technology acceptance model
TIB: theory of interpersonal behavior
TRA: theory of reasoned action
