# Supplementary material for: Nurses’ Intention to Integrate AI Into Their Practice: Survey Study in Canada
Source: JMIR Nurs. 2025 Sep 5;8:e76795. doi: 10.2196/76795 (PMC12413143; doi:10.2196/76795)
Supplement: Multimedia Appendix 1 [file nursing-v8-e76795-s001.docx]

**Experience**

*Please specify your years of experience as a nurse (check one)*

1= 1-5 years

2= 6-10 years

3= 11-15 years

4= 16-20 years

5= 21-30 years

6= 30+ years

9= Prefer not to respond

**Gender**

*Please specify your gender (check one)*

0= Male

1= Female

9= Prefer not to respond

**Digital technology literacy**

*What is your level of familiarity with consumer digital technologies (mobile applications on smartphones or tablets, applications available on personal computers)? (1=Very low and 5=Very high)*

**Familiarity with AI**

*What is your level of familiarity with AI-based technologies in general? (check one) (1=No familiarity at all and 10=Extremely high level of familiarity)*

**Role of AI in job and profession**

*What impact do you believe the use of AI has already had or will have on various aspects related to your job and profession? (1=Very negative impact and 5=Very positive impact)*

- Tasks or responsibilities related to my profession
- The decisions I make (clinical judgment, diagnosis, etc.)
- Best clinical practices related to my profession
- Working conditions of the members of my profession
- Productivity of the staff in my profession
- Interest of new professionals in my profession
- Patients' trust in my work and professional judgment
- Retention rate of professionals in my profession

**Trust in AI**

*To what extent do you agree or disagree with the following statements? (1=Strongly disagree and 5=Strongly agree)*

- I trust AI-based technologies
- I am not afraid of using AI-based technologies
- The opacity of some AI-based technologies does not affect my overall trust in AI

**Perceived impactfulness of AI**

*I consider the use of AI-based technologies in my clinical practice to be... (check one per line)*

| Useless | 1 | 2 | 3 | 4 | 5 | Useful |
| --- | --- | --- | --- | --- | --- | --- |
| Harmful | 1 | 2 | 3 | 4 | 5 | Beneficial |
| Irresponsible | 1 | 2 | 3 | 4 | 5 | Responsible |
| Bad | 1 | 2 | 3 | 4 | 5 | Good |
| Not at all stimulating | 1 | 2 | 3 | 4 | 5 | Extremely stimulating |
| Stressful | 1 | 2 | 3 | 4 | 5 | Reassuring |

**Anxiety towards AI**

*Please answer each of the following questions (1=Yes and 0=No)*

- Are you anxious about the new practical skills you need to learn to use AI in your work?
- Does the idea of being replaced by AI in your work make you anxious?
- Does the extent of the changes caused by AI in your profession make you anxious?
- Do you feel anxious or stressed when the topic of AI is discussed in general?

**Intention to integrate AI into practice**

*To what extent to you agree or disagree with the following statements? (1=Strongly disagree and 5=Strongly agree)*

- I intend to participate in training sessions, conferences, or workshops in the future to familiarize myself with AI or to improve my knowledge in this area
- I am open to the idea of using AI-based technologies in my practice
- I would like to integrate AI into my future professional practice
